# VExD: a curated resource for human gene expression alterations following viral infection

**DOI:** 10.1093/g3journal/jkad176

**Published:** 2023-08-02

**Authors:** Phillip J Dexheimer, Mario Pujato, Krishna M Roskin, Matthew T Weirauch

**Affiliations:** Division of Biomedical Informatics, Cincinnati Children's Hospital, Cincinnati, OH 45229, USA; Department of Biomedical Informatics, University of Cincinnati College of Medicine, Cincinnati, OH 45221, USA; Division of Biomedical Informatics, Cincinnati Children's Hospital, Cincinnati, OH 45229, USA; Division of Biomedical Informatics, Cincinnati Children's Hospital, Cincinnati, OH 45229, USA; Division of Immunobiology, Cincinnati Children's Hospital, Cincinnati, OH 45229, USA; Department of Pediatrics, University of Cincinnati College of Medicine, Cincinnati, OH 45221, USA; Department of Pediatrics, University of Cincinnati College of Medicine, Cincinnati, OH 45221, USA; Center for Autoimmune Genomics and Etiology, Cincinnati Children's Hospital, Cincinnati, OH 45229, USA; Divisions of Human Genetics, Biomedical Informatics and Developmental Biology, Cincinnati Children's Hospital, Cincinnati, OH 45229, USA

**Keywords:** virus–host interaction, gene expression, web resource, human virus, RNA-seq, microarray, functional genomics‌

## Abstract

Much of the host antiviral response is mediated through changes to host gene expression levels. Likewise, viruses induce changes to host gene expression levels in order to promote the viral life cycle and evade the host immune system. However, there is no resource that specifically collects human gene expression levels pre- and post-virus infection. Further, public gene expression repositories do not contain enough specialized metadata to easily find relevant experiments. Here, we present the Virus Expression Database (VExD), a freely available website and database, that collects human gene expression datasets in response to viral infection. VExD contains ∼8,000 uniformly processed samples obtained from 289 studies examining 51 distinct human viruses. We show that the VExD processing pipeline captures known antiviral responses in the form of interferon-stimulated genes. We further show that the datasets collected in VExD can be used to quickly identify supporting data for experiments performed in human cells or model organisms. VExD is freely available at https://vexd.cchmc.org/.

## Introduction

Viral infection can have profound and lasting impacts on human health and society (Vos *et al*. 2020). Viruses affect host gene expression levels by altering transcriptional mechanisms in infected cells and by initiating the host's immune response. Numerous transcriptomic studies of human viral infection have been deposited in public databases such as the gene expression omnibus (GEO) (Barrett *et al*. 2013). However, descriptions of samples and studies in GEO are freeform text and require either manual curation or machine learning to search at a large scale (Wang, Lachmann *et al*. 2019). This task is particularly complicated in virus-based studies due to the inconsistent nomenclature of viruses in the literature (Gibbs 2003). As a recent example, the virus that causes COVID-19 was named in the earliest publications as “Wuhan coronavirus” (Liu and Saif 2020), then “2019-nCov” (Xie and Chen 2020), before finally settling into the accepted name of “severe acute respiratory syndrome-related coronavirus 2" (Gorbalenya *et al*. 2020).

In addition to using different nomenclature for the same virus, different studies employ different experimental assays and analysis pipelines to quantify gene expression levels and changes between conditions. These differing conditions make comparison between studies difficult since true biological differences often cannot be distinguished from technical or analytical artifacts.

Collectively, these issues greatly impede progress on studies of the human response to virus infection. To the best of our knowledge, the only resource that attempts to provide human gene expression signatures in response to infection by a variety of viruses is Harmonizome (Rouillard *et al*. 2016). One of the datasets in that resource (https://maayanlab.cloud/Harmonizome/dataset/GEO+Signatures+of+Differentially+Expressed+Genes+for+Viral+Infections) contains 366 signatures of viral infection. However, these signatures cover only 15 distinct viruses, no attempt was made to standardize virus nomenclature, and only limited information is provided regarding data provenance. Other immunology genomics resources exist, such as ImmPort (Bhattacharya *et al*. 2018), HIPC Dashboard (Smith *et al*. 2022), and ImmuneSpace (Diray-Arce *et al*. 2022). However, these resources collect data with a much broader scope and only rarely contain gene expression data following viral infection.

**Fig. 1.**
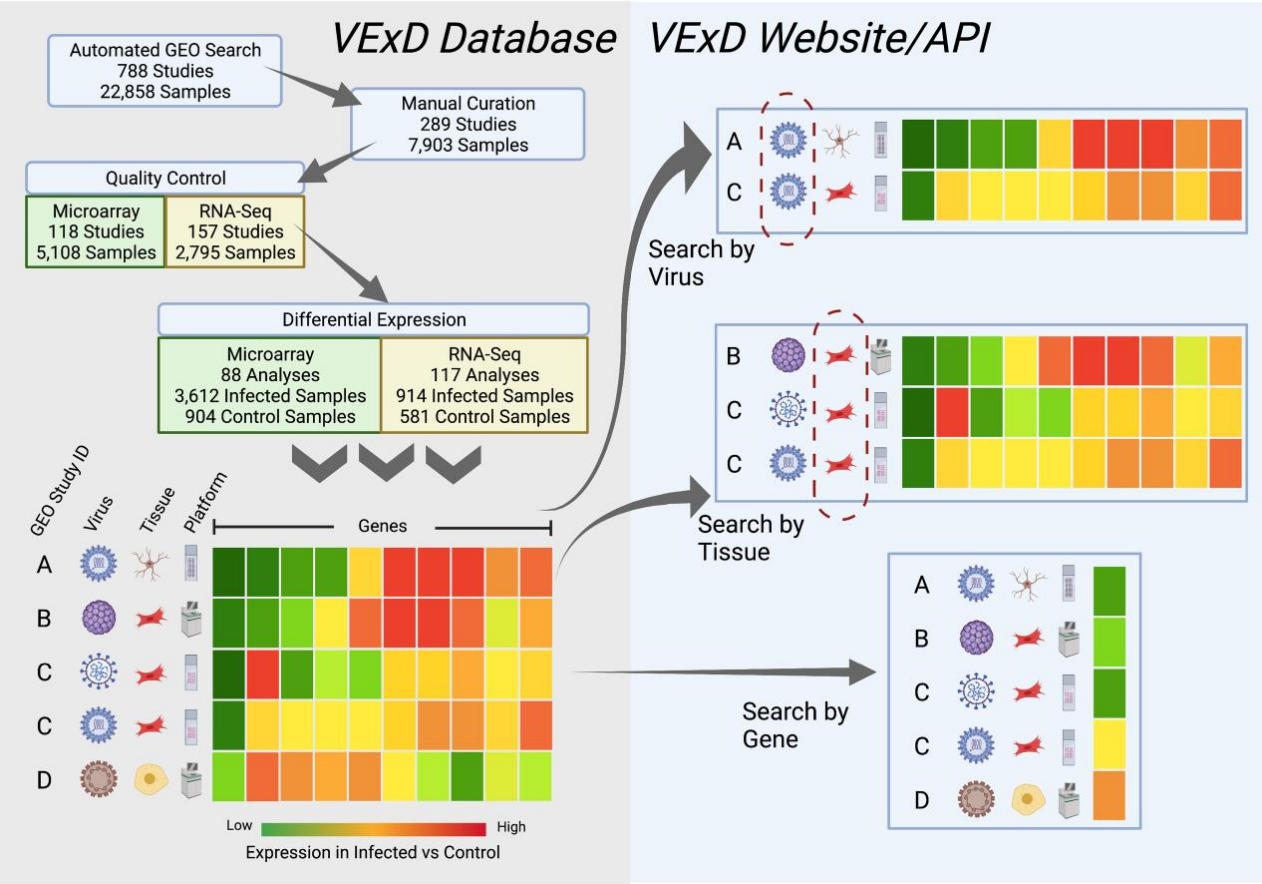
Overview of the VExD methods and website. The underlying database (left side) contains differential expression results for all viral infections of human cells or tissues found in the GEO. Results are annotated with the source GEO ID, the virus species, the cell/tissue type (using the BTO), and the experimental platform used in the original study. Users of the VExD website (right side) can search the results by virus or tissue type or can search by gene to see a global view of gene expression in response to viral infection. Static database dumps are available for download, and an extensive API is available for programmatic access. Figure created with BioRender.com.

To address the need for uniformly annotated and processed gene expression data from human cells infected with viruses, we present VExD, the Virus Expression Database (Fig. 1). VExD contains a manually curated list of 7,903 samples from 289 gene expression studies of infection by 51 distinct viruses. All studies have been subjected to uniform quality control and data processing to maximize comparability across studies. The VExD web interface is easily browsable and searchable and provides automatically generated figures for comparing human gene expression levels across studies and conditions. VExD data are available for download in multiple convenient formats and through an application programming interface (API). VExD is a unique and accessible resource for globally and uniformly assessing the effect of human viruses on gene expression levels.

## Methods

### Identification of samples

We assembled a list of viruses known to infect humans based on annotations in ViralZone (Hulo *et al*. 2011), UniProt (The UniProt Consortium 2019), the Virus-Host DB (Mihara *et al*. 2016), and literature curation studies (Taylor *et al*. 2001; Woolhouse *et al*. 2012), as well as common synonyms and abbreviations. Following the taxonomy published by the International Committee on Taxonomy of Viruses (Walker *et al*. 2020), we standardized all viruses to the level of species. Our final list (Supplementary Table 1) comprises 332 human-infecting species.

Using a tool that we developed for a previous publication (Harley *et al*. 2018), we searched GEO for any studies mentioning a virus name or abbreviation in the study or sample description. Only samples annotated as human were considered. To enable standardized processing, we also required studies that use either the Affymetrix microarray or Illumina bulk RNA-sequencing platforms. The 22,858 samples identified with the automated scanner were then manually curated to remove false-positive results (Fig. 1, top left). Samples were removed for a variety of reasons, including: (1) wrong platform, (2) use of a virus as a molecular biology tool rather than an infection (e.g. to immortalize cells or as a gene promoter), or (3) the virus name abbreviation identified by the scanner was not used in a virology context (e.g. measles virus = MV = megavolts). The remaining 7,903 samples were annotated with BRENDA tissue ontology (BTO) terms representing the assayed cell type or tissue (Gremse *et al*. 2011) and were loaded into a Mongo database.

### Data quality control

RNA-Seq samples were quantified using kallisto (Bray *et al*. 2016) with the “--bias” parameter to account for sequence-specific biases. For paired-end samples, kallisto was allowed to derive the fragment length distribution from the data (default behavior). For single-end samples, the distribution was specified as having a mean length of 300 nt and an SD of 30 nt (parameters “-l 300 -s 30”). For all samples, the reference transcriptome was the Ensembl human gene set (version 102). Final gene-level quantities were obtained by summing the transcripts per million values for all associated transcripts. Samples with too few usable reads (pseudoaligned reads <1.5 million) or poor overall alignment (<18% reads pseudoaligned) were removed.

For microarray studies, BrainArray version 25 Chip Definition Files (CDF) (Dai *et al*. 2005) that remap probes onto Ensembl v102 genes were used. All microarrays of a single array type were analyzed together using robust multichip analysis (RMA) (Irizarry *et al*. 2003) as implemented in the Affymetrix Power Tools. Samples were removed if the residual error in the RMA model was too large (mad_residual_mean > 0.80) or if the overall signal was too low (pm_mean < 65).

For RNA-seq and microarray platforms, studies with fewer than 60% of their samples passing these criteria were removed. In total, 234 of 2,129 (11%) RNA-seq samples and 186 of 4,964 (3.7%) microarray samples were removed by these quality control steps.

### Differential expression quantification

VExD contains differential expression results between comparable virus-infected samples and uninfected controls, allowing users to easily determine the human gene expression changes observed for each virus. Comparable samples are defined as those coming from the same study, using the same tissue or cell type, and the same technological platform, with at least 2 infected samples and 2 uninfected control samples. A total of 205 differential expression analyses involving 4,526 infected samples and 1,485 uninfected controls are available in VExD (Fig. 1). All tests used a 2-sided Welch's *t-*test with unequal variance and Benjamini–Hochberg correction.

### Gene enrichment analysis

When analyzing gene sets, we compare the fold changes observed for the genes in the set across all experiments in VExD with all other gene observations. Specifically, we employ the Brunner–Munzel test (Brunner and Munzel 2000; Neubert and Brunner 2007) to determine whether the distribution of values associated with the gene set is significantly different from the remaining values in VExD. The Brunner–Munzel test is a generalization of the Wilcoxon Mann–Whitney test that does not assume equal variance within each population.

The stochastic superiority statistic is called the “common language effect size” in some sources and is calculated as described in (Brunner *et al*. 2018), where it is named $\hat{\;p}$.

### VExD website and API

The VExD website was created with the Python Flask framework and was served using the Apache webserver with the mod_wsgi module. Users can search by any combination of virus and tissue type to view the overall gene expression levels or search for individual genes across all studies by symbol, alias, or Ensembl ID (Fig. 1). Results pages display the virus, cell type, fold change, and adjusted *P*-value for a given gene in all studies. Download links provide study and differential expression information in both a tab-delimited text file and a compressed, machine-readable format (Apache Parquet). VExD also supports an API that can be used to programmatically query the database on the command line.

## Results

### Identification of virus infection samples

We sought to identify and collate a comprehensive collection of experiments that show gene expression differences in human cells after infection with a virus. GEO is a large repository of gene expression experiments, making it a natural source for our collection efforts. Before identifying experiments in GEO that involve viral infection, we first assembled a comprehensive list of viruses known to infect humans (Supplementary Table 1, details in *Methods*). We then searched all GEO sample records for any virus name, alias, or abbreviation and manually curated the results for our final set of 7,903 samples in 289 experiments of viral infection of human cells (further details in *Methods*). In addition to verifying that the samples underwent viral infection (or were a matched uninfected control), we standardized both the virus name and the cell or tissue type of the sample. Viruses were named according to the species name defined by the International Committee on Taxonomy of Viruses (Walker *et al*. 2020). For cell/tissue identification, samples were annotated with the most relevant term from the BTO (Gremse *et al*. 2011).

### Uniform data processing and quality control

Because the data in question span ∼17 years of genomic technology and analysis improvements, we developed a uniform data processing and quality control pipeline. Using updated definition files from the BrainArray project (Dai *et al*. 2005), we discarded outdated microarray probes and used current definitions of genes across all samples for both microarray and RNA-Seq experiments. Briefly, RNA-Seq samples were quantified with kallisto (Bray *et al*. 2016) using Ensembl gene definitions, and microarrays were processed with RMA (Irizarry *et al*. 2003) using BrainArray custom CDF files that use the same gene definitions as the RNA-Seq samples. Following quality control (see *Methods*), 3.7% of microarray samples and 11% of RNA-Seq samples were removed. Differential analysis comparing gene expression levels between infected and uninfected cells within a study was performed using Welch's *t*-test.

### The VExD web portal

The complete results of these curation and uniform processing efforts constitute VExD, freely available at https://vexd.cchmc.org. VExD offers the ability to search for experiments by virus and/or tissue type and allows the download of differential expression results for each experiment. In addition, users can search for individual genes or gene sets to examine their relative expression levels across all experiments contained in VExD. The Downloads page allows users to download the complete list of GEO experiments within VExD, the complete differential expression results across all experiments, or differential expression results for each virus. Finally, computational users can make use of the API of VExD to perform any search available on the website from the command line or their own programming environment.

### Validation

**Fig. 2.**
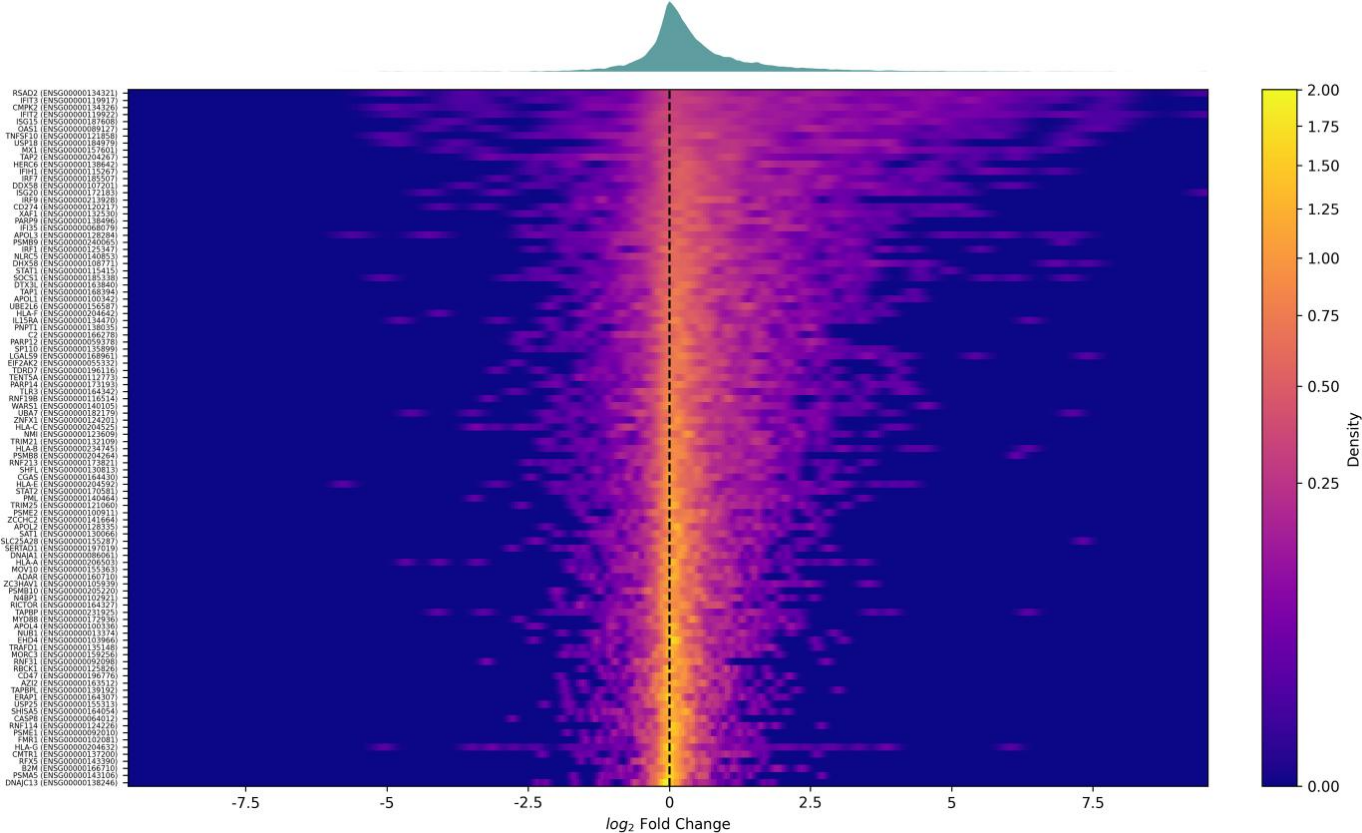
Pan-VExD expression of the core ISGs identified in (Shaw *et al*. 2017). Each row of the heatmap represents all differential expression results in VExD for a single gene, with colors indicating the density estimate of observed fold changes for that gene. The *x*-axis shows the log_2_-transformed fold change of infected samples vs their matched controls. A density plot of all 98 genes is shown above the heatmap. Note that both the heatmap and density plot have a greater signal on the right side, indicating a global increase in gene expression, as expected for ISGs. Randomly selected genes do not produce this shift (Supplementary Fig. 1).

We sought to validate the VExD data processing methodology. We first examined the behavior of the 98 core interferon-stimulated genes (ISGs) that Shaw *et al.* (2017) identified as conserved across vertebrates (Fig. 2). Since ISGs are broadly activated in response to viral infection, we expected to see these genes largely upregulated in VExD. Indeed, we find that the stochastic superiority statistic for ISGs—that is, the probability that a randomly selected ISG measurement is larger than a randomly selected non-ISG measurement—is 64.7%, compared with a random expected value of 50%. Further, ISG fold changes are significantly distinct from all other (non-ISG) genes (*P*-value <10^−30^, 2-sided Brunner–Munzel test). Conversely, random selection of 98 genes shows no substantial change (Supplementary Fig. 1: stochastic superiority statistic of 49.6%, *P*-value =0.17), as expected. The VExD website includes functionality (https://vexd.cchmc.org/enrich) to test input gene sets for enrichment.

**Fig. 3.**
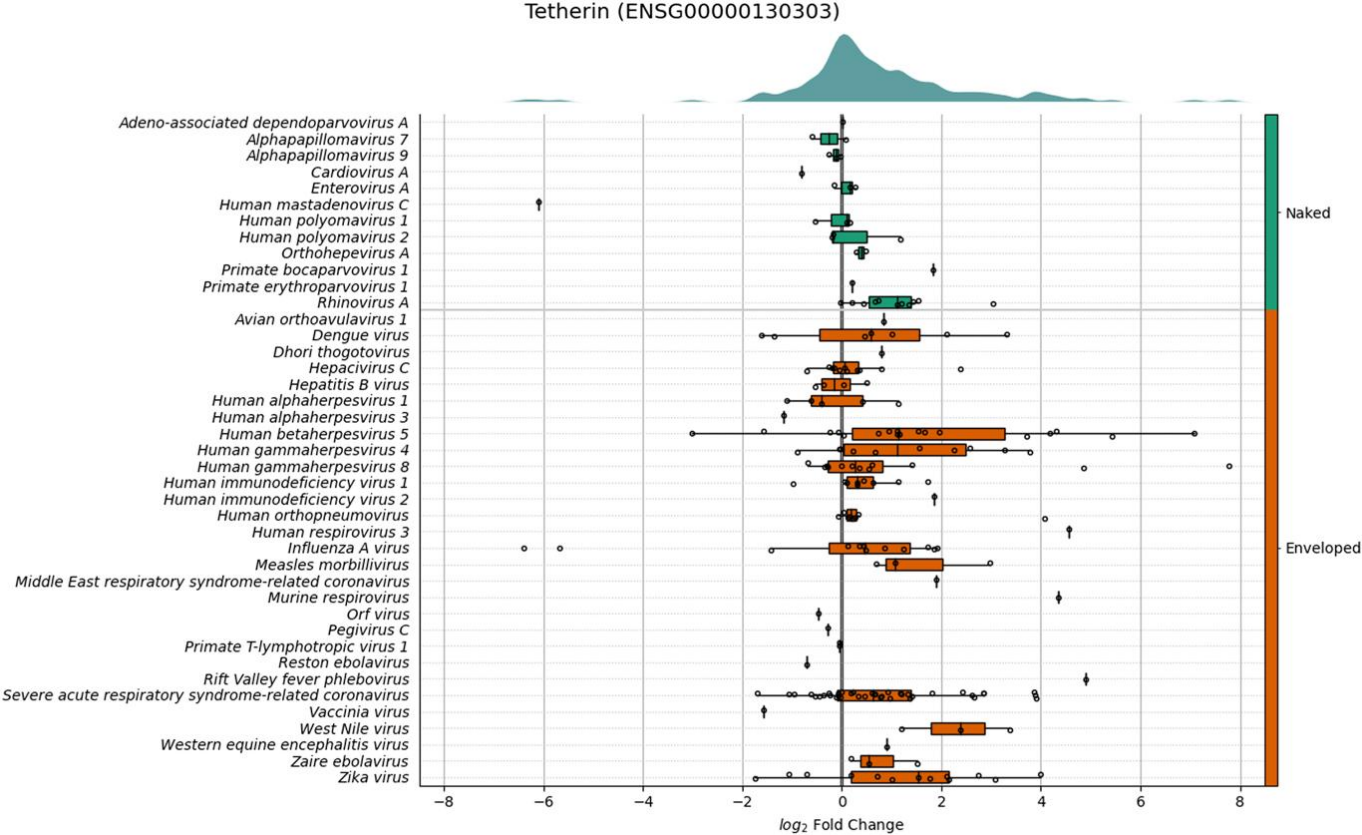
Expression of tetherin (*BST2*) across all experiments in VExD. Experiments are stratified by virus (rows), which are then ordered by whether the virion has an envelope or not (right side). Within each virus, the log_2_ fold change of tetherin in each distinct experiment is plotted as a dot. Box-and-whisker overlays are included as well. Broadly, tetherin is overexpressed in response to enveloped viruses but is not overexpressed for naked (unenveloped) viruses, with the (novel) exception of rhinoviruses.

We next examined the ability of VExD data to highlight virus-specific effects. Tetherin is an ISG that physically binds virions to the host cell, preventing their export (Neil *et al*. 2008). It has been confirmed to be effective against a wide variety of enveloped viruses, including retroviruses (Neil *et al*. 2008), rhabdoviruses (Weidner *et al*. 2010), and filoviruses (Kaletsky *et al*. 2009). Indeed, when we examine the expression of tetherin in the experiments contained in VExD (Fig. 3), we see that it is more likely to be overexpressed in response to infection with an enveloped virus than a virus with no envelope. The stochastic superiority statistic for tetherin expression after infection with an enveloped virus is 66.6%, with a Brunner–Munzel *P*-value of 5 × 10^−10^. Conversely, after infection with a naked (nonenveloped) virus, the stochastic superiority statistic is only 61.8%, with a *P*-value of 0.04. Strikingly, tetherin expression is uniformly upregulated in all 11 experiments of *Rhinovirus A* infection in VExD, even though rhinovirus does not have an envelope. When considering only the nonrhinovirus naked viruses, the stochastic superiority statistic falls to 49.5%, with a *P*-value of 0.9.

**Fig. 4.**
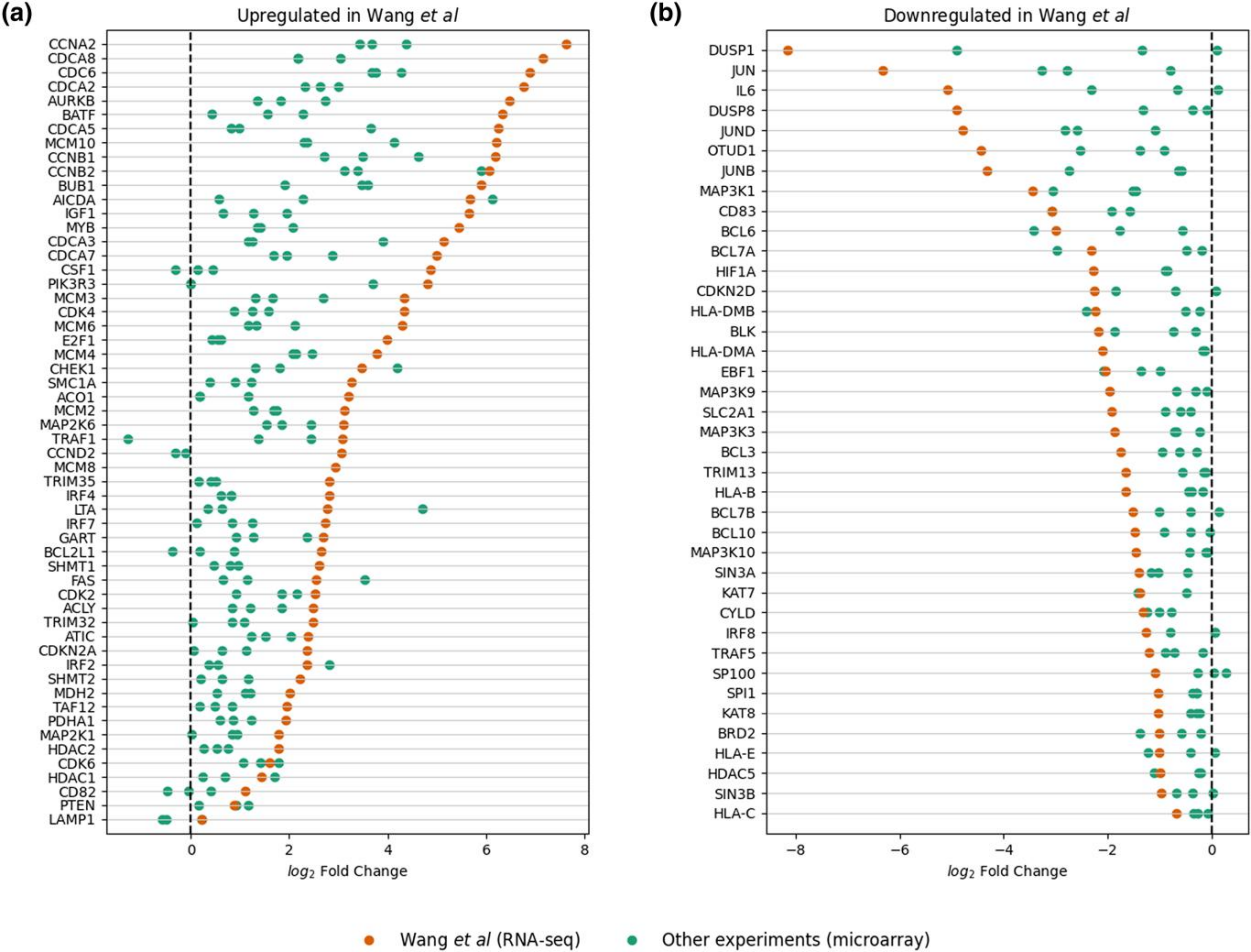
Differentially expressed genes identified by (Wang, Li *et al*. 2019) show consistent expression across all similar experiments in VExD. In each panel, the genes identified by Wang *et al.* are displayed in rows. Each dot represents the fold change of that gene in a single VExD experiment, after restricting to experiments of Epstein–Barr virus infection in human B cells. Results from Wang *et al.* are highlighted. a) Genes found to be upregulated in Wang *et al*. and b) genes found to be downregulated in Wang *et al*.

We next examined the consistency of VExD results across experiments. A recent study (Wang, Li *et al*. 2019) performed a time series of Epstein–Barr virus (EBV, *Human gammaherpesvirus 4*) infections against primary human B cells. As such, this experiment is included in VExD, along with 3 prior experiments that also used infected B cells with EBV. Notably, Wang *et al.* used RNA-seq to assess gene expression differences, while the other 3 experiments in VExD used microarrays. When we assess the differentially expressed genes highlighted in Fig. 1 of Wang *et al.*, we see that the direction of regulation is maintained in the other EBV-infected B-cell experiments contained in VExD (Fig. 4). These results are consistent with previous observations of the concordance between RNA-seq and microarray technologies (Wang *et al*. 2014; Rao *et al*. 2019), although the experiments in this case were performed in different laboratories with different experimental protocols.

**Fig. 5.**
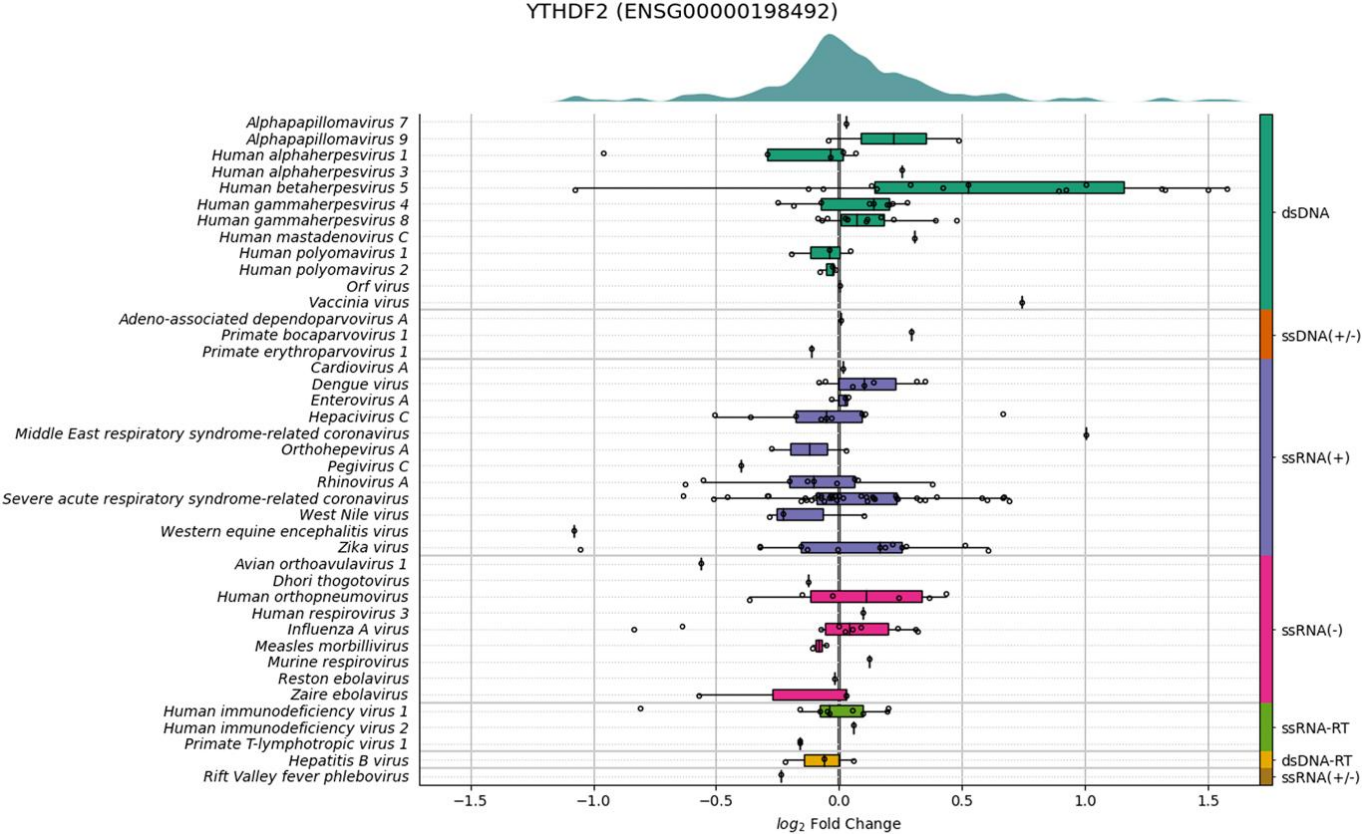
Expression of *YTHDF2* across all VExD experiments. Experiments are stratified by virus (rows), which are then ordered by genome type (right side). Within each virus, the log_2_ fold change of *YTHDF2* in each distinct experiment is plotted as a dot. Box-and-whisker overlays are also included. Note that *YTHDF2* is strongly upregulated in response to *Human betaherpesvirus 5* (CMV) infections, but not other viruses.

Finally, we used VExD to add context to findings obtained from a model organism. A recent publication (Ma *et al*. 2021) demonstrated that the m^6^A reader gene *Ythdf2* is overexpressed in mouse natural killer cells after infection with murine cytomegalovirus (*Murid betaherpesvirus 1*). Using VExD, we find that the human ortholog *YTHDF2* is also overexpressed in response to human cytomegalovirus (CMV, *Human betaherpesvirus 5*) infections, but not to other virus types (Fig. 5). In addition, although none of the VExD experiments were performed in natural killer cells, a tissue-specific response is apparent, with only fibroblasts and epithelium showing upregulation among the cell types assayed (Supplementary Table 2).

## Discussion

We present VExD, a comprehensive, curated resource of transcriptomic studies of viral infection in human cells. VExD contains 7,903 samples from 289 experiments, all processed through uniform quality control, quantification, and differential expression pipelines. The VExD website (https://vexd.cchmc.org/) presents these results in a simple searchable form, as a bulk download, or via an API. In addition, the VExD website allows users to query the response of genes to a viral infection, stratified by virus and tissue type through real-time data visualizations.

We demonstrate the utility of VExD using 4 case studies. In the first, we show that the interferon response is highly consistent across viruses, as expected (Fig. 2). In the second (Fig. 3), we demonstrate the ability of VExD to highlight virus-specific effects by showing that the gene expressing tetherin is specifically upregulated in enveloped viruses. In our third case study (Fig. 4), we show that the results in VExD are consistent across experiments, laboratories, and analytic platforms. Specifically, the genes identified by Wang, Li *et al*. (2019) as significantly regulated by EBV in B cells show similar patterns in 3 independent experiments in VExD, despite the Wang group using RNA-seq and the other experiments using microarray. In our final case study (Fig. 5), we examine a report of a novel antiviral gene (*Ythdf2*) found in a model organism (mouse). Using the data contained in VExD, we find that the human ortholog is also upregulated in humans in a virus- and cell-type specific manner. In this manner, the breadth of experimental data captured by VExD provides a broader context to investigators, allowing for more nuanced hypothesis generation.

VExD is a novel and extensive resource that will be of broad benefit to the community. However, it does have some limitations. First, VExD is limited to the studies that have been deposited in GEO, which means that particular viruses or cell types may be underrepresented. In addition, although we performed extensive quality control of the transcriptomic assays deposited in GEO before including them (see *Methods*), we could not verify all experimental details, such as whether the appropriate multiplicity of infection or cell type was assayed. As such, it is possible that VExD contains experiments that do not represent successful infections. Second, VExD relies upon manual curation of automatically generated GEO search results. While this is a labor-intensive process that is susceptible to human error, in our experience, a fully automated process does not adequately capture the complex descriptions contained in GEO.

To our knowledge, VExD is the largest available resource of gene expression data following viral infection. Using the website or the API, investigators can use VExD to identify relevant transcriptomic studies, query gene expression levels in a system of interest, or investigate the relevance of individual genes or gene sets in the antiviral response. As new virus gene expression data continue to become available, we anticipate that future updates to VExD will continue to improve its utility through the incorporation of these additional datasets.

## Web resources

The VExD website (https://vexd.cchmc.org) is freely available, with no registration required. Differential expression results can be downloaded from VExD on a per-study or per-virus basis or all in a single file. Data curation results are also available for download. Source code for the website is available at https://github.com/pdexheimer/vexd.

## Supplementary Material

jkad176_Supplementary_Data

## Data Availability

Figure 2 and Supplementary Fig. 1 in this paper can be regenerated from the “Enrichment” tab of the website. Figure 5 can be regenerated from the “Genes” tab of the website. Supplementary File S1 contains a source code to recreate Fig. 3. Supplementary File S2 contains a source code to recreate Fig. 4. The genes used in Fig. 4a were the “genes of interest” from groups 1, 2, and 3 (upregulated upon infection) from Fig. 1 of (Wang, Li *et al*. 2019). The genes used in Fig. 4b were similarly chosen from groups 7 and 8 of the same figure. Groups 4, 5, and 6 showed more complicated, time-dependent expression patterns and were thus not relevant for a simple “infected vs control” comparison. Supplementary Table 3 contains gene lists used in all figures. Supplemental material available at G3 online.

## References

[jkad176-B1] Barrett T , Wilhite SE, Ledoux P, Evangelista C, Kim IF, Tomashevsky M, Marshall KA, Phillippy KH, Sherman PM, Holko M, et al NCBI GEO: archive for functional genomics data sets–update. Nucleic Acids Res. 2013;41(Database issue):D991–D995. doi:10.1093/nar/gks1193. 23193258 PMC3531084

[jkad176-B2] Bhattacharya S , Dunn P, Thomas CG, Smith B, Schaefer H, Chen J, Hu Z, Zalocusky KA, Shankar RD, Shen-Orr SS, et al ImmPort, toward repurposing of open access immunological assay data for translational and clinical research. Sci Data. 2018;5(1):180015. doi:10.1038/sdata.2018.15. 29485622 PMC5827693

[jkad176-B3] Bray NL , Pimentel H, Melsted P, Pachter L. Near-optimal probabilistic RNA-Seq quantification. Nat Biotechnol. 2016;34(5):525–527. doi:10.1038/nbt.3519. 27043002

[jkad176-B4] Brunner E , Bathke AC, Konietschke F. Rank and Pseudo-Rank Procedures for Independent Observations in Factorial Designs. Springer International Publishing; 2018. p. 75–180.

[jkad176-B5] Brunner E , Munzel U. The nonparametric Behrens-Fisher problem: asymptotic theory and a small-sample approximation. Biom J. 2000;42(1):17–25. doi:10.1002/(SICI)1521-4036(200001)42:1<17::AID-BIMJ17>3.0.CO;2-U.

[jkad176-B6] Dai M , Wang P, Boyd AD, Kostov G, Athey B, Jones EG, Bunney WE, Myers RM, Speed TP, Akil H, et al Evolving gene/transcript definitions significantly alter the interpretation of GeneChip data. Nucleic Acids Res. 2005;33(20):e175. doi:10.1093/nar/gni179. 16284200 PMC1283542

[jkad176-B7] Diray-Arce J , Miller HER, Henrich E, Gerritsen B, Mulè MP, Fourati S, Gygi J, Hagan T, Tomalin L, Rychkov D, et al The immune signatures data resource, a compendium of systems vaccinology datasets. Sci Data. 2022;9(1):635. doi:10.1038/s41597-022-01714-7. 36266291 PMC9584267

[jkad176-B8] Gibbs AJ . Viral nomenclature, where next? Arch Virol. 2003;148(8):1645–1653. doi:10.1007/s00705-003-0150-5. 12898338

[jkad176-B9] Gorbalenya AE , Baker SC, Baric RS, de Groot RJ, Drosten C, Gulyaeva AA, Haagmans BL, Lauber C, Leontovich AM, Neuman BW, et al The species severe acute respiratory syndrome-related coronavirus: classifying 2019-nCoV and naming it SARS-CoV-2. Nat Microbiol. 2020;5(4):536–544. doi:10.1038/s41564-020-0695-z. 32123347 PMC7095448

[jkad176-B10] Gremse M , Chang A, Schomburg I, Grote A, Scheer M, Ebeling C, Schomburg D. The BRENDA tissue ontology (BTO): the first all-integrating ontology of all organisms for enzyme sources. Nucleic Acids Res. 2011;39(Database issue):D507–D513. doi:10.1093/nar/gkq968. 21030441 PMC3013802

[jkad176-B11] Harley JB , Chen X, Pujato M, Miller D, Maddox A, Forney C, Magnusen AF, Lynch A, Chetal K, Yukawa M, et al Transcription factors operate across disease loci, with EBNA2 implicated in autoimmunity. Nat Genet. 2018;50(5):699–707. doi:10.1038/s41588-018-0102-3. 29662164 PMC6022759

[jkad176-B12] Hulo C , de Castro E, Masson P, Bougueleret L, Bairoch A, Xenarios I, Le Mercier P. ViralZone: a knowledge resource to understand virus diversity. Nucleic Acids Res. 2011;39(suppl_1):D576–D582. doi:10.1093/nar/gkq901. 20947564 PMC3013774

[jkad176-B13] Irizarry RA , Hobbs B, Collin F, Beazer-Barclay YD, Antonellis KJ, Scherf U, Speed TP. Exploration, normalization, and summaries of high density oligonucleotide array probe level data. Biostatistics. 2003;4(2):249–264. doi:10.1093/biostatistics/4.2.249. 12925520

[jkad176-B14] Kaletsky RL , Francica JR, Agrawal-Gamse C, Bates P. Tetherin-mediated restriction of filovirus budding is antagonized by the Ebola glycoprotein. Proc Natl Acad Sci USA. 2009;106(8):2886–2891. doi:10.1073/pnas.0811014106. 19179289 PMC2650360

[jkad176-B15] Liu S-L , Saif L. Emerging viruses without borders: the Wuhan coronavirus. Viruses. 2020;12(2):130. doi:10.3390/v12020130. 31979013 PMC7077218

[jkad176-B16] Ma S , Yan J, Barr T, Zhang J, Chen Z, Wang L-S, Sun JC, Chen J, Caligiuri MA, Yu J. The RNA m6A reader YTHDF2 controls NK cell antitumor and antiviral immunity. J Exp Med. 2021;218(8):e20210279. doi:10.1084/jem.20210279. 34160549 PMC8225680

[jkad176-B17] Mihara T , Nishimura Y, Shimizu Y, Nishiyama H, Yoshikawa G, Uehara H, Hingamp P, Goto S, Ogata H. Linking virus genomes with host taxonomy. Viruses. 2016;8(3):66. doi:10.3390/v8030066. 26938550 PMC4810256

[jkad176-B18] Neil SJD , Zang T, Bieniasz PD. Tetherin inhibits retrovirus release and is antagonized by HIV-1 Vpu. Nature. 2008;451(7177):425–430. doi:10.1038/nature06553. 18200009

[jkad176-B19] Neubert K , Brunner E. A studentized permutation test for the non-parametric Behrens–fisher problem. Comput Stat Data Anal. 2007;51(10):5192–5204. doi:10.1016/j.csda.2006.05.024.

[jkad176-B20] Rao MS , Van Vleet TR, Ciurlionis R, Buck WR, Mittelstadt SW, Blomme EAG, Liguori MJ. Comparison of RNA-Seq and microarray gene expression platforms for the toxicogenomic evaluation of liver from short-term rat toxicity studies. Front Genet. 2019;9:636. doi:10.3389/fgene.2018.00636. 30723492 PMC6349826

[jkad176-B21] Rouillard AD , Gundersen GW, Fernandez NF, Wang Z, Monteiro CD, McDermott MG, Ma’ayan A. The harmonizome: a collection of processed datasets gathered to serve and mine knowledge about genes and proteins. Database. 2016;2016:baw100. doi:10.1093/database/baw100. 27374120 PMC4930834

[jkad176-B22] Shaw AE , Hughes J, Gu Q, Behdenna A, Singer JB, Dennis T, Orton RJ, Varela M, Gifford RJ, Wilson SJ, et al Fundamental properties of the mammalian innate immune system revealed by multispecies comparison of type I interferon responses. Malik H, editor. PLoS Biol. 2017;15(12):e2004086. doi:10.1371/journal.pbio.2004086. 29253856 PMC5747502

[jkad176-B23] Smith KC , Chawla DG, Dhillon BK, Ji Z, Vita R, Van Der Leest EC, Weng JYJ, Tang E, Abid A, The Human Immunology Project Consortium (HIPC), et al A curated collection of human vaccination response signatures. Sci Data. 2022;9(1):678. doi:10.1038/s41597-022-01558-1. PMC9643367 36347894

[jkad176-B24] Taylor LH , Latham SM, Woolhouse ME. Risk factors for human disease emergence. Philos Trans R Soc Lond B Biol Sci. 2001;356(1411):983–989. doi:10.1098/rstb.2001.0888. 11516376 PMC1088493

[jkad176-B25] The UniProt Consortium . UniProt: a worldwide hub of protein knowledge. Nucleic Acids Res. 2019;47(D1):D506–D515. doi:10.1093/nar/gky1049. 30395287 PMC6323992

[jkad176-B26] Vos T , Lim SS, Abbafati C, Abbas KM, Abbasi M, Abbasifard M, Abbasi-Kangevari M, Abbastabar H, Abd-Allah F, Abdelalim A, et al Global burden of 369 diseases and injuries in 204 countries and territories, 1990–2019: a systematic analysis for the global burden of disease study 2019. Lancet. 2020;396(10258):1204–1222. doi:10.1016/S0140-6736(20)30925-9. 33069326 PMC7567026

[jkad176-B27] Walker PJ , Siddell SG, Lefkowitz EJ, Mushegian AR, Adriaenssens EM, Dempsey DM, Dutilh BE, Harrach B, Harrison RL, Hendrickson RC, et al Changes to virus taxonomy and the statutes ratified by the international committee on taxonomy of viruses (2020). Arch Virol. 2020;165(11):2737–2748. doi:10.1007/s00705-020-04752-x. 32816125

[jkad176-B28] Wang C , Gong B, Bushel PR, Thierry-Mieg J, Thierry-Mieg D, Xu J, Fang H, Hong H, Shen J, Su Z, et al The concordance between RNA-seq and microarray data depends on chemical treatment and transcript abundance. Nat Biotechnol. 2014;32(9):926–932. doi:10.1038/nbt.3001. 25150839 PMC4243706

[jkad176-B29] Wang Z , Lachmann A, Ma’ayan A. Mining data and metadata from the gene expression omnibus. Biophys Rev. 2019;11(1):103–110. doi:10.1007/s12551-018-0490-8. 30594974 PMC6381352

[jkad176-B30] Wang C , Li D, Zhang L, Jiang S, Liang J, Narita Y, Hou I, Zhong Q, Zheng Z, Xiao H, et al RNA sequencing analyses of gene expression during Epstein-Barr virus infection of primary B lymphocytes. J Virol. 2019;93(13):e00226-19. doi:10.1128/JVI.00226-19. 31019051 PMC6580941

[jkad176-B31] Weidner JM , Jiang D, Pan X-B, Chang J, Block TM, Guo J-T. Interferon-induced cell membrane proteins, IFITM3 and tetherin, inhibit vesicular stomatitis virus infection via distinct mechanisms. J Virol. 2010;84(24):12646–12657. doi:10.1128/JVI.01328-10. 20943977 PMC3004348

[jkad176-B32] Woolhouse M , Scott F, Hudson Z, Howey R, Chase-Topping M. Human viruses: discovery and emergence. Philos Trans R Soc Lond B Biol Sci. 2012;367(1604):2864–2871. doi:10.1098/rstb.2011.0354. 22966141 PMC3427559

[jkad176-B33] Xie M , Chen Q. Insight into 2019 novel coronavirus—an updated interim review and lessons from SARS-CoV and MERS-CoV. Int J Infect Dis. 2020;94:119–124. doi:10.1016/j.ijid.2020.03.071. 32247050 PMC7118633

