# Supplementary material for: VExD: a curated resource for human gene expression alterations following viral infection
Source: G3 (Bethesda). 2023 Aug 2;13(10):jkad176. doi: 10.1093/g3journal/jkad176 (PMC10542171; doi:10.1093/g3journal/jkad176)
Supplement: jkad176_Supplementary_Data [file jkad176_supplementary_data.zip › Supplemental_Material_Legends_G3-2023-404433.docx]

# Supplemental Material Legends

**Figure S1** – Pan-VExD expression of 98 randomly-selected genes. As in Figure 2, each row represents the fold change of a single gene across all experiments within VExD, and the top panel contains a density plot of all 98 genes. As expected, these genes are largely unchanged in response to viral infection. The VExD website includes functionality to regenerate this figure with different random genes or user-selected gene sets.

**Table S1** – The species name, aliases, and abbreviations for all human-infecting viruses eligible for inclusion in VExD.

**Table S2** – Per-experiment details for *YTHDF2* expression following cytomegalovirus (*Human betaherpesvirus 5*) infection, highlighting the cell type-specific nature of this gene’s regulation. This table is a subset of the information available from <https://vexd.cchmc.org/gene?q=ENSG00000198492> or the results endpoint of the VExD API.

**Table S3** – The genes used in Figures 2, 4, and S1 of this paper.

**File S1** – the Python source code used to generate Figure 3 of this paper.

**File S2** – the Python source code used to generate Figure 4 of this paper.
