# Supplementary figures and images for: VExD: a curated resource for human gene expression alterations following viral infection
Source: G3 (Bethesda). 2023 Aug 2;13(10):jkad176. doi: 10.1093/g3journal/jkad176 (PMC10542171; doi:10.1093/g3journal/jkad176)

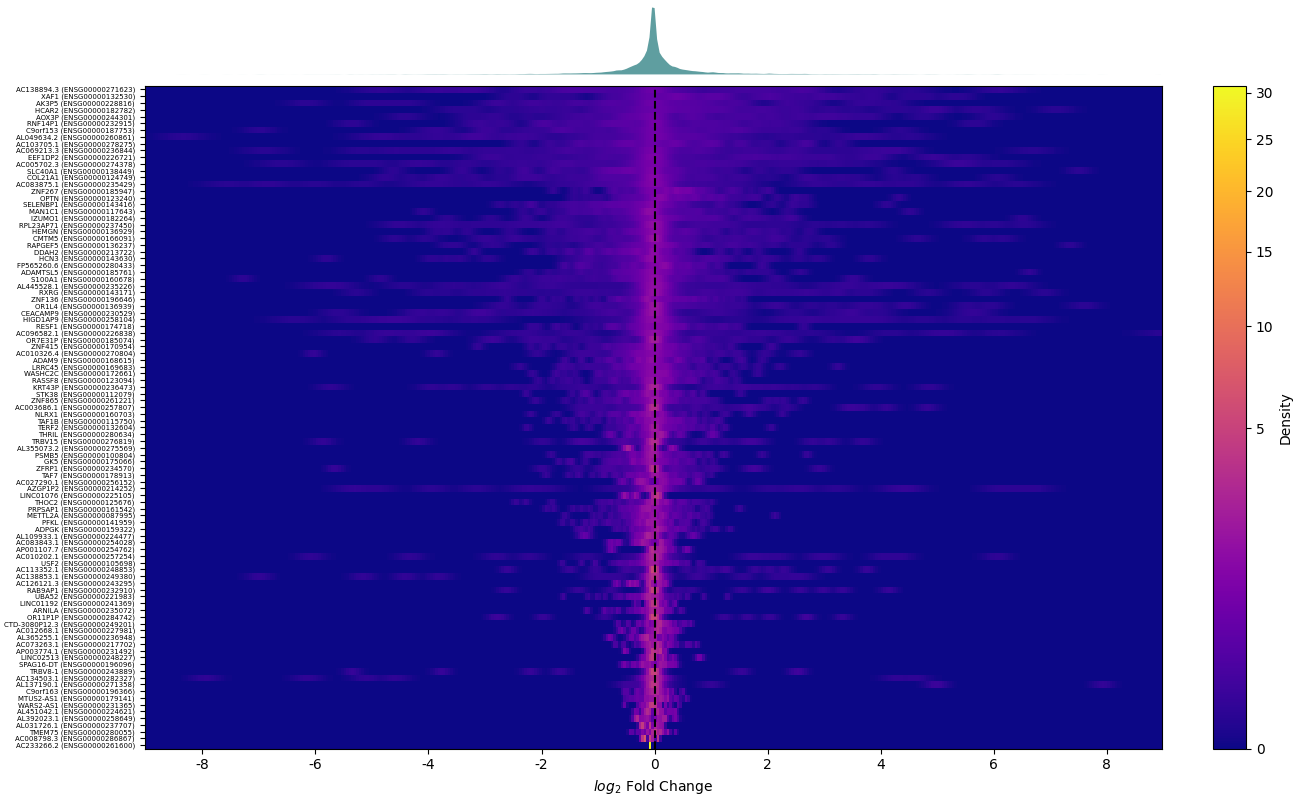

Supplement: jkad176_Supplementary_Data [file jkad176_supplementary_data.zip › Figure_S1_G3-2023-404433.png]
